# Cyp26a1 supports postnatal retinoic acid homeostasis and glucoregulatory control

**DOI:** 10.1016/j.jbc.2023.104669

**Published:** 2023-04-01

**Authors:** Hong Sik Yoo, Michael A. Cockrum, Joseph L. Napoli

**Affiliations:** Graduate Program in Metabolic Biology, Nutritional Sciences and Toxicology, UC-Berkeley, Berkeley, California, USA

**Keywords:** corticosterone, *Cyp26a1*, glucagon, glucoregulation, insulin, retinoic acid

## Abstract

Considerable evidence confirms the importance of Cyp26a1 to all-*trans*-retinoic acid (RA) homeostasis during embryogenesis. In contrast, despite its presence in postnatal liver as a potential major RA catabolizing enzyme and its acute sensitivity to induction by RA, some data suggested that Cyp26a1 contributes only marginally to endogenous RA homeostasis postnatally. We report reevaluation of a conditional *Cyp26a1* knockdown in the postnatal mouse. The current results show that *Cyp26a1* mRNA in WT mouse liver increases 16-fold upon refeeding after a fast, accompanied by an increased rate of RA elimination and a 41% decrease in the RA concentration. In contrast, *Cyp26a1* mRNA in the refed homozygotic knockdown reached only 2% of its extent in WT during refeeding, accompanied by a slower rate of RA catabolism and no decrease in liver RA, relative to fasting. Refed homozygous knockdown mice also had decreased Akt1 and 2 phosphorylation and pyruvate dehydrogenase kinase 4 (*Pdk4*) mRNA and increased glucokinase (*Gck*) mRNA, glycogen phosphorylase (Pygl) phosphorylation, and serum glucose, relative to WT. Fasted homozygous knockdown mice had increased glucagon/insulin relative to WT. These data indicate that Cyp26a1 participates prominently in moderating the postnatal liver concentration of endogenous RA and contributes essentially to glucoregulatory control.

All-*trans*-retinoic acid (RA), a metabolite of vitamin A (retinol), contributes to postnatal regulation of energy metabolism by preventing pre-adipocyte differentiation into mature white adipocytes, by stimulating lipolysis and by promoting fatty acid oxidation (1, 2, 3, 4, 5, 6). RA dosing or reduction decreases or increases adiposity, respectively. RA also exerts glucoregulatory control by regulating endocrine pancreatic cell differentiation and by opposing insulin actions, including through inducing gluconeogenesis (7). Multiple metabolic reactions regulate RA tissue concentrations (8). These include retinol dehydrogenation into retinal, catalyzed by retinol dehydrogenases, reduction of retinal catalyzed by retinal reductases, dehydrogenation of retinal into RA, and retinol esterification to limit substrate available for RA biosynthesis. Insulin retards RA biosynthesis through expelling FoxO1 from the nucleus and diminishing the mRNA of retinol and retinal dehydrogenases (9). This permits maximum insulin action during feeding, while maximizing RA actions during fasting.

Catabolism also contributes to RA homeostasis. Inhibiting catabolism with “conazoles” boosted RA potency and/or inhibited catabolism in cultured cells, providing early evidence that degradation tempers RA effects (10, 11, 12). Discovery of Cyp26a1 (aka P450RA1), as the founder of a unique family of cytochromes P450, identified enzymes specific to RA clearance (13, 14, 15). Several investigations have reported Cyp26a1 activities during mouse embryogenesis (16, 17, 18, 19). *Cyp26a1* ablation causes embryonic lethality by mid to late gestation. Major defects include spina bifida and malformations of the kidneys, the urogenital tract, the hindgut, and abnormal neural differentiation. Atypical induction of *Cyp26a1* in mouse embryonic stem cells introduces defects in lineage differentiation (20). Ablation of the retinal dehydrogenase *Raldh2* rescues *Cyp26a1*-null mice substantially, indicating the importance of balancing RA concentrations by coordinating biosynthesis and catabolism (21). Also consistent with coordinate modulation of RA biosynthesis and catabolism, *Cyp26a1* and the retinol dehydrogenase *Rdh1* display inversely related mRNA expression in embryos (e7.5 to e18.5) and liver (e12.5 to P2M) (1). In postnatal rat liver, RA and vitamin A induce liver *Cyp26a1* expression potently and relatively rapidly, with a greater dynamic range than other potential P450s that catabolize RA (22). Even so, contributions of Cyp26a1 to postnatal RA homeostasis seem less certain. Postnatal ablation of *Cyp26a1* in Sertoli and germ cells revealed no involvement in RA function during spermatogenesis (23). A conditional, postnatal, whole-body *Cyp26a1* knockout did not alter RA serum and tissue concentrations or produce consistent adverse phenotypes (24). *Cyp26a1*-null mice, however, had disrupted retinoid-induced myeloid hematopoiesis. These data fostered the conclusion that Cyp26a1 has no significant influence in regulating endogenous RA signaling. *Cyp26a1* knockout, however, decreased the elimination rate of a toxic RA dose (10 mg/kg) in mouse plasma by 6-fold, prompting the conclusion that Cyp26a1 contributes significantly only to clearing exogenous RA overdose.

We have reported that glucagon and cortisol secreted during fasting decrease *CYP26A1* mRNA, whereas refeeding increases its mRNA (25). The decrease in *CYP26A1* mRNA occurred *via* the glucocorticoid receptor binding to the RARα coactivation complex, hampering its transcriptional activity. Inhibition of RA catabolism during fasting, a phase of optimum RA biosynthesis, helps maintain high RA concentrations. By accelerating RA catabolism, the increase in Cyp26a1 during refeeding complements insulin action in arresting RA biosynthesis. These data suggest an essential contribution of Cyp26a1 to RA’s regulation of postnatal energy balance and provide additional evidence that RA and energy balance relate closely to each other. The present work aimed to extend insight into the contribution of liver Cyp26a1, because human, rat, and mouse livers express *CYP26A1/Cyp26a1* intensely, but express *CYP26B1/Cyp26b1* and *CYP26C1/Cyp26c1* weakly (26, 27, 28, 29). Here, we further examine two issues: the contribution(s) of Cyp26a1 to regulating liver RA concentrations and the physiological importance of Cyp26a1. We report that *Cyp26a1* contributes substantially to moderating the endogenous liver RA concentration in postnatal mice, and its ablation disrupts glucoregulatory control.

## Results

**Figure 1:**
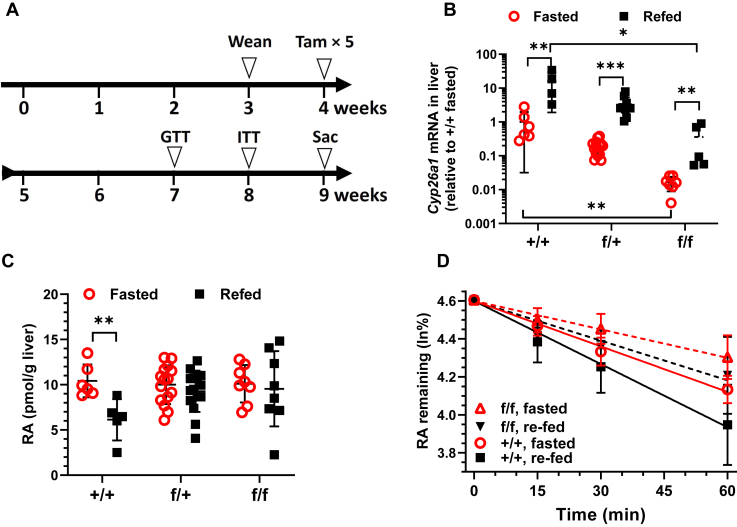
**Cyp26a1 knockdown prevents the decrease in liver RA in response to feeding.***A*, protocol for creating and evaluating all strains of mice. All genotypes were treated identically. *B*, *Cyp26a1* mRNA in livers (n = 5–15). *C*, quantification of liver RA by LC/MS/MS (n = 5–15). *D*, microsomal RA catabolism, elimination t½ were determined by quantifying by LC/MS/MS the ln % remaining of 50 pmol RA with the time of microsomal incubation. *Cyp26a1* WT, +/+; WT/Cre+ heterozygous, f/+; *Cyp26a1* homozygous floxed and Cre+, f/f. *Red open circles*: fasted 16 h; *black filled squares*: refed 6 h following fasting 16 h. RA, retinoic acid. ∗∗*p* < 0.01; ∗∗∗*p* < 0.001.

The current experiments relied on a conditional knockdown (KD) of *Cyp26a1* and a purified diet that contained sufficient but not copious amounts of vitamin A. *Cyp26a1*-floxed tamoxifen (tam)-inducible Cre mice were administered tam at 4-weeks-old for five consecutive days (Fig. 1*A*). Glucose tolerance (GTT) and insulin tolerance tests (ITT) were done at 7- and 8-weeks-old, respectively, on the same mice. Serum and liver tissues were harvested at 9-weeks-old following a 16 h fast or upon 6 h refeeding after a 16 h fast.

*Cyp26a1* mRNA in WT mouse liver responded with a 16-fold increase to 6 h refeeding after an overnight fast (Fig. 1*B*). Tam-induced Cre-driven KD decreased *Cyp26a1* mRNA in fasted heterozygous and homozygous mice to 19% and 1.6% of WT, respectively. After refeeding, *Cyp26a1* mRNA increased to only ∼21 and 2% of WT in heterozygous and homozygous KD mice, respectively. Consistent with previous observations, refeeding prompted a 41% decrease in WT liver RA (9, 25) (Fig. 1*C*). Increased liver RA during fasting and decreased liver RA during refeeding serves its function as an anti-insulin autacoid (7). In contrast to WT, neither the heterozygous nor the homozygous KD mouse liver decreased RA after refeeding. *Cyp26a1* KD also decreased the rate of RA elimination by liver microsomes (Fig. 1*D*). WT liver microsomes eliminated RA at half-lives of 89 and 65 min during fasting and refeeding, respectively. The t½ elimination rates increased to 141 and 110 min in homozygous fasted and refed KD mice, respectively. Neither retinyl esters nor retinol in liver varied by fasting *versus* refeeding or with genotypes (Fig. S1, *A* and *B*). We also confirmed that serum RA does not reflect liver RA, as published previously (Fig. S1*C*) (30).

No apparent signs of gross liver damage presented in H&E-stained sections of any genotype, such as necrosis, steatosis, or fibrosis (Fig. S2). Glycogen deposits appeared in refed groups but did not differ by genotype (Fig. S3). Inflammation lesions occurred in two of four homozygous KD mice examined (Fig. S4, *A* and *B*). Although they were minimal to mild, the lesions accompanied yellow-green deposits of the iron-containing protein hemosiderin, distinguishable from spontaneous inflammation foci observed in WT liver sections (Fig. S4, *C*–*E*).

**Figure 2:**
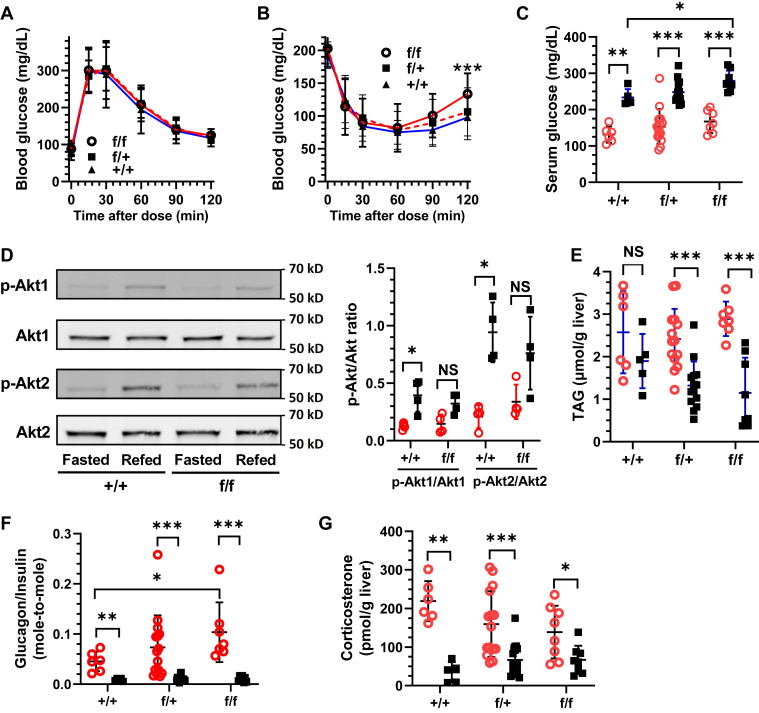
**Cyp26a1 deficiency attenuates insulin signaling.***A*, glucose tolerance tests (n = 10–30 mice). *B*, insulin tolerance tests (n = 10–30 mice). *C*, serum glucose at 9 weeks old after 16 h fasting or 6 h refeeding after a 16 h fast (n = 6–15 mice). *D*, Western blots of pAkt1/Akt1 and pAkt2/Akt2 (n = 4 mice). *E*, hepatic triacylglycerol (TAG) was determined by a colorimetric ELISA (n = 6–14 mice). *F*, ratio of serum glucagon/insulin (mole-to-mole, n = 5–15 mice). *G*, quantification of corticosterone in liver by LC/MS/MS (n = 5–15 mice). Fasted, *red circles*; refed *black squares*. ∗p<0.05, ∗∗p<0.01, ∗∗∗ p<0.001.

GTTs did not differ among genotypes (Fig. 2*A*). Upon an insulin challenge (ITT), glucose levels in each genotype decreased at similar rates (Fig. 2*B*). After 60 min, however, serum glucose levels began to diverge, eventually reaching a statistically significant increase in homozygous KD relative to WT. Serum glucose increased 46 mg/dl more in refed homozygous KD mice than WT (Fig. 2*C*). We hypothesized that the elevated glucose resulted from an increase in hepatic glucose production, indicating insulin resistance in liver. Western blots of Akt serine/threonine kinase 1 and 2 (Akt1 and Akt2) *versus* phosphorylated Akt1 and Akt2 showed that fasting increased phosphorylation of both Akt1 and 2 in WT mice, as expected, by >3-fold and 4.5-fold, respectively (Fig. 2*D*). The degrees of Akt1 and Akt2 phosphorylation between fasted and refed homozygous KD mice were blunted (∼2-fold each) and did not reach statistical significance (p ∼ 0.06). Liver triacylglycerol (TAG) did not differ significantly in WT fasting *versus* refed mice. TAG in livers of refed KD mice was significantly lower than in fasted mice, with homozygous KD mice attaining only 61% of the level in fasted WT mice (Fig. 2*E*). The glucagon/insulin ratio in fasted homozygous KD increased ∼2.3-fold more than WT (Fig. 2*F*), driven by the glucagon increase in the homozygote KD during fasting (Fig. S5). Corticosterone increased >6-fold during fasting in WT mice but only ∼2-fold in KD mice (Fig. 2*G*).

**Figure 3:**
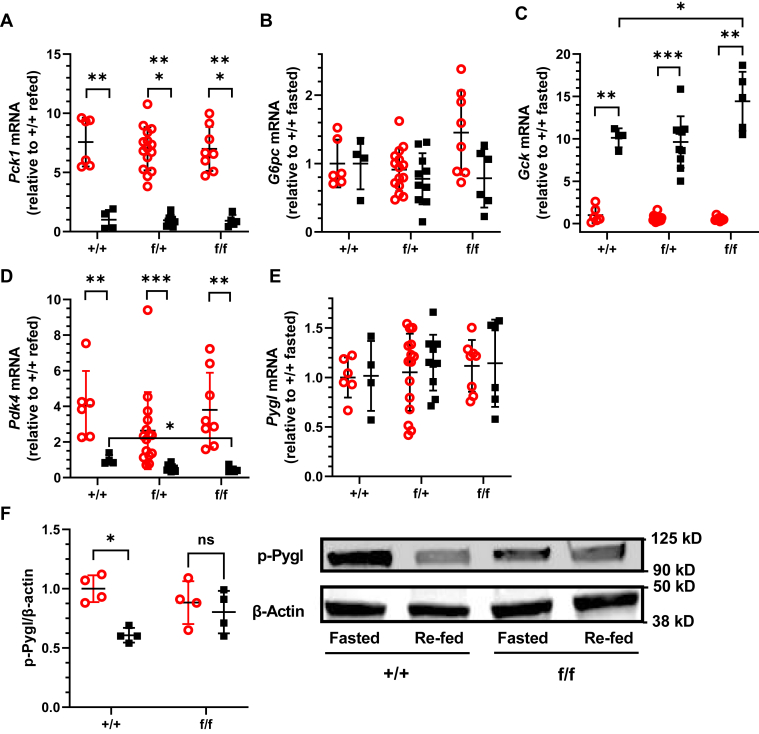
**Effects of Cyp26a1 knockdown on glucoregulation.***A*, *Pck1* mRNA. *B*, *G6pc* mRNA. *C*, *Gck* mRNA. *D*, *Pdk4* mRNA. *E, Pygl* mRNA. *F*, Western blots of phosphorylated Pygl (p-Ser15). All n = 5 to 15 mice, except Pygl Western blot, where n = 4 mice each genotype, each dietary condition. *Red circles*, fasted 16 h; *black squares*, 6 h refed after 16 h fasted. ∗p<0.05, ∗∗p<0.01, p<0.001.

Phosphorylated Akt phosphorylates the FoxO1 transcription factor, which causes its nuclear export and proteolysis, resulting in decreased expression of phosphoenolpyruvate carboxykinase (*Pck1*) and glucose-6 phosphatase (*G6pc*) (31). *Pck1* expression increased during fasting, as expected, but did not differ among genotypes, during fasting or after refeeding (Fig. 3*A*). *G6pc* expression increased ∼1.5-fold during fasting in homozygous KD relative to WT but did not attain statistical significance (p ∼ 0.08). (Fig. 3*B*). *Gys2* mRNA, which encodes the rate-limiting enzyme of glycogen synthesis, did not change with genotype during fasting or refeeding (Fig. S6). *Gck* mRNA increased in all genotypes with refeeding but extended 1.4-fold higher in refed homozygous KD mice relative to WT (Fig. 3*C*), consistent with the increased serum glucose (Fig. 2*C*). Fasting induces *Pdk4* expression, which inhibits the pyruvate dehydrogenase complex and increases pyruvate for gluconeogenesis (32). Fasting increased *Pdk4* mRNA, but no differences occurred by genotype (Fig. 3*D*). In contrast, *Pdk4* mRNA in refed homozygous KD mice reached only 46% of the level in WT mice. This suggests a shift during refed homozygous KD mice from sparing pyruvate to generating acetyl-CoA. Glycogen phosphorylase (*Pygl*) mRNA, which encodes the rate-limiting enzyme of liver glycogenolysis, also did not change with fasting *versus* refeeding or with genotype (Fig. 3*E*). Insulin induced dephosphorylation and inactivation of phospho-Pygl in WT, but not in the homozygous KD mice (Fig. 3*F*).

## Discussion

This work addresses two issues primarily: actions of Cyp26a1 in regulating the liver RA concentration, and because Cyp26a1 activity links critically to the physiological functions of RA, we determined contributions of Cyp26a1 to glucoregulatory control. Decreases in *Cyp26a1* mRNA in heterozygous and homozygous KD mice accompanied 56% increases in liver RA during refeeding relative to WT mice, consistent with a major contribution of liver Cyp26a1 to regulating RA. Changes in the RA elimination t½ provide additional insight. These values show that in addition to Cyp26a1 exerting a major impact during refeeding, it also does so during fasting. For example, in WT mice, the RA elimination t½ increased by 25 min during fasting relative to refeeding. In the homozygous KD, this value increased by 40 min during fasting relative to refeeding. Moreover, fasted homozygous KD mice had an extended RA elimination t½ of 52 min relative to fasted WT mice. Therefore, Cyp26a1 contributes to the catabolism of endogenous RA in liver during both fasting and refeeding. Lack of an increase in liver RA concentrations during fasting in KD *versus* WT mice likely indicates regulatory mechanisms other than Cyp26a1 activity. RA homeostasis reflects actions of cellular retinol-binding proteins, multiple retinol and retinal dehydrogenases, retinal reductases, retinol esterification enzymes, and retinyl ester hydrolases, besides catabolic enzymes (33). Another tier of regulation involves positive feedback by RA to induce lecithin:retinol acyltransferase to limit the retinol concentration. Overall, these data indicate a complex process during fasting, which involves more than *Cyp26a1* expression and activity, while affirming the contribution of liver Cyp26a1 to regulating RA.

Multiple metabolic intermediates and hormones regulate hepatic glucose production (34, 35, 36). The glucagon/insulin ratio exerts enormous influence, as do corticoids. An increase in the glucagon/insulin ratio in the fasted KD mice reflects insulin resistance and would increase gluconeogenesis, hepatic glucose secretion, and/or glycogenolysis (37). Although RA regulates *Pck1* and *G6pc* transcriptionally (38, 39), RA did not increase measurably during fasting in the homozygous KD mouse, accounting for their lack of increase. Corticosterone, another inducer of *G6pc*, did not decrease significantly during fasting in KD, perhaps accounting for the lack of a significant difference in *G6pc* (40).

Most metabolic differences between WT and homozygous KD mice occurred during refeeding, consistent with the increased RA concentration in KD mice. The increase during refeeding of serum glucose suggests insulin resistance, as manifested by decreased Akt phosphorylation and the increase in serum glucose. The serum glucose increase, along with the increase in RA, likely induced the increase in liver *Gck* mRNA, a gene transcriptionally regulated by RA (41). The decrease in *Pdk4* would allow an increase in activity of the pyruvate dehydrogenase complex, thereby decreasing pyruvate and gluconeogenesis and providing additional acetyl-CoA for entry into the TCA cycle. The relative TAG decreases in the liver of fed KD mice indicate insulin resistance, the increase in the glucagon/insulin ratio, and/or the increase in RA. Apparently, the overall increase in blood glucose and the decrease in TAG represents the sum of counter balancing controls. The increase in all genotypes in *Pck1* mRNA reveals synergistic induction by RA and glucocorticoids (42). A lack of an increase in *Pck1* during fasting in the KD mice relative to WT, when corticoids and RA attain maximum levels, perhaps indicates maximum induction had occurred. No data indicate that RA regulates *Gys2* and *Pyg1* mRNA, accounting for the lack of transcriptional responses in these genes. Pyg1, however, undergoes insulin-induced dephosphorylation and inactivation after feeding and glucagon-induced phosphorylation and activation during fasting (43). The lack of a decrease in Pyg1 phosphorylation in the refed KD mouse reflects the impact of the increased glucagon/insulin ratio on the liver and would contribute to the increased serum glucose.

Zhong *et al*. (24) reported a decrease in erythropoiesis in KD mice, which might underlie the liver hemosiderin deposits observed in this report. Liver hemosiderin results from excess iron deposited from blood iron or from ferritin breakdown (44). The Zhong report concluded Cyp26a1 has minor effects on postnatal RA homeostasis and contributes only to catabolism of exogenous RA. We have generated data to the contrary, but our experimental protocol contrasts in several essential properties with those in the Zhong report. The latter fed a commercial grain-based chow diet with copious vitamin A (20 IU vitamin A with 1.5 ppm β-carotene/g diet, followed by 30 IU vitamin A/g diet). Chow diets with copious vitamin A have improved or rescued phenotypes of three retinoid-related gene KOs, *Rdh1*, *Rbp4*, and *Crbp2* (1, 45, 46). The purified diet used in the current study contained 4 IU vitamin A/g, as recommended by the National Research Council for rodents (47, 48). Vitamin A exerts hormesis, that is, it wields concentration-dependent effects. In addition, grain-based diets contain multiple cereal grains, which vary seasonally, and harbor mycotoxins, heavy metals, and other pollutants, absent in purified diets (49). Grain-based diets also contain phytoestrogens that function as estrogen receptor modifiers. Crosstalk between RA and estrogen occurs and modifies the actions of each (5, 6). Purified diets, as used here, do not rely on grain for carbohydrates, but on cornstarch, dextrin, and sucrose. Finally, glucoregulatory mechanisms were not evaluated in the Zhong report, which assessed ad lib–fed mice. Ad lib–fed mice occur individually in a dietary status between fasted and fed and would have RA levels varying individually between the two energy states. The major variables of evaluating ad lib–fed mice, feeding diets copious in vitamin A, and not assessing glucoregulatory control amply account for the differences in conclusions.

The data herein affirm a contribution of Cyp26a1 to RA homeostasis postnatally and further show that Cyp26a1 contributes to glucoregulatory control. Inhibition of RA metabolism could provide an alternative to RA dosing. RA metabolism–blocking agents (aka RAMBA), that is, inhibitors of Cyp26 isomers, have been evaluated for therapeutic use to increase endogenous RA concentrations (50, 51). This approach represents an attempt to increase tissue RA, while avoiding the toxic effects of RA dosing, the short half-life of dosed RA, and autoinduction of RA degradation. RAMBA have been used for treating dermatological diseases, such as acne, psoriasis, and ichthyosis, and have undergone clinical trials for treating prostate and breast cancer, with limited effects. RAMBA developed so far do not seem selective for inhibiting RA catabolism, which also limits their use. The current report suggests another cautionary note for RAMBA use: potential disruption of glucoregulatory control.

## Experimental procedures

### Mice

Inducible *Cyp26a1*-deficient male mice were generated by crossing the tamoxifen-inducible Cre line, B6.129-*Gt(ROSA)26Sor*^*tm1(cre/ERT2)Tyj*^/J (strain# 008463), purchased from The Jackson Laboratory, with *Cyp26a1*-floxed mice donated by Dr Martin Petkovich (16). Three experimental groups (*Cyp26a1* +/+, *Cyp26a1* f/+, *Cyp26a1* f/f) were produced by F1 x F1 crossing (*Cyp26a1* f/+ with tam Cre+/−). All mice were administered tam once daily by i.p. injection (75 mg/kg) at 4-weeks-old for five consecutive days, as reported previously (16, 23, 24). No mice succumbed to tam dosing. Mice were fed a purified AIN93G diet (Research Diets, D10012G) containing 4 IU retinyl acetate/g and 7% fat. Serum and liver samples were collected from 9-weeks-old mice fasted 16 h and compared to those refed 6 h after a 16 h fast, after anesthesia in an isoflurane chamber, followed by cervical dislocation. Blood samples, drawn from the vena cava, were allowed to clot on ice and centrifuged 30 min at 12,000*g* and 4 °C. Sera and livers were snap-frozen in liquid nitrogen and stored at −80 °C until assayed. Animal experimental protocols were approved by the University of California Berkeley Animal Care and Use Committee.

### GTTs and ITTs

GTTs were done at 7-weeks-old. ITTs) were done at 8-weeks-old on the same mice (Fig. 1*A*). Mice were fasted 16 h (5 PM-9 AM) before the GTT and 4 h in the morning before the ITT and housed in individual cages without food, but with free access to water. Either glucose (2 g/kg body weight) or insulin (0.5 IU/kg body weight) was injected I.P. Blood glucose from tail tips was measured using an Ascensia CONTOUR NEXT glucometer.

### H&E staining of liver sections

Segments ∼1.5 cm long and 3 mm thick were cut from the middle of the left lobe and fixed in 10% neutral buffered formalin (Sigma HT501128) for 3 days. Formalin-fixed, paraffin-embedded liver segments were cut into 8-μm sections. Deparaffinized sections were stained with H&E. Tissue sections were imaged using a Zeiss Axio Imager M1 microscope equipped with QImaging 5MPix MicroPublisher.

### Retinoid quantification

Retinyl esters and retinol were quantified by LC/UV (52). RA was quantified by LC/MS/MS, following a published method with the modification that liver was homogenized in methanol and centrifuged 5 min at 1200*g* to remove precipitates (25).

### Liver microsomal assays

Liver samples (∼100 mg) were homogenized in buffer (10 mM Tris–HCl, 250 mM sucrose, pH 7.4) using a glass pestle tissue grinder on ice. The homogenate was centrifuged 10 min at 10,000*g* and 4 °C. The supernatant was ultracentrifuged 1 h at 100,000*g* and 4 °C. The microsomal pellet was resuspended in assay buffer (150 mM KCl, 5 mM MgCl_2_, 20 mM Tris–HCl, pH 7.4). RA catabolism was assessed at 37 °C. The 0.5 ml incubation mixture contained the assay buffer, 50 pmol RA, an NADPH regenerating system (5 units of glucose-6-phosphate dehydrogenase, 500 nmol NADP+, 500 nmol glucose 6-phosphate), and 50 μg microsomal protein. The reaction was terminated by adding 2 ml methanol.

### Western blots

Proteins in liver samples were extracted with 1 ml RIPA buffer (Thermo Fisher Scientific, 89901), which included protease and phosphatase inhibitors (Thermo Fisher Scientific, A32961). The Bradford assay determined total protein concentrations. Thirty micrograms of protein were mixed 1:1 with Laemmli sample buffer (Bio-Rad, 1610737), including 5% β-mercaptoethanol, heated 5 min at 95 °C, and cooled on ice. Protein samples were separated by 12% SDS-PAGE (Bio-Rad, 4561044), transferred onto nitrocellulose membranes (Bio-Rad, 1620115), which were blocked in Intercept (TBS) blocking buffer (LI-COR, 927-60001), and immunoblotted overnight at 4 °C with antibodies against Akt1 (Cell Signaling, D9R8K), phospho(S473)-Akt1 (Cell Signaling, D7F10), Akt2 (Cell Signaling, 5B5), phospho(S474)-Akt2 (Cell Signaling, D3H2), phospho-(S15)-Pygl (Thermo Fisher Scientific, PA5-114628), and β-actin (Abcam, ab8226). Primary antibodies were diluted 1:2000. Near-infrared fluorescent dye-conjugated secondary antibodies were as follows: anti-rabbit (LI-COR, 926-32211) and anti-mouse (LI-COR, 926-68070) at 1:5000. Immunoblots were developed with a LICOR Odyssey Imaging System. Western blot band density was quantified by integrating areas under curves using ImageJ 1.53. Antibodies for phosphor-Akt1 and Akt2 were stripped by shake-incubating in Restore PLUS Western blot stripping buffer (Thermo Fisher Scientific, 46430) at 37 °C for 10 min, followed by reblock and immunoblotting with the Akt1 and 2 antibodies. Signals were normalized to actin. For p-Pygl, actin-normalized signals were then normalized to the average of WT fasted set as 1. Data are provided in Table S1.

### Gene expression assays

Total RNA was isolated using TRI Reagent (Sigma, T9424), quantified using a ThermoScientific NanoDrop One, and reverse-transcribed using iScript cDNA Synthesis Kit (Bio-Rad, 1708891). Quantitative PCR was performed with a Bio-Rad CFX Connect Real-Time Detection System using PrimeTime Gene Expression Master Mix (Integrated DNA Technologies (1055772). Gene expression was analyzed by the ΔΔ-Ct method, normalized to *Gusb*, and expressed as fold change relative to controls. Primers are listed in Table S2.

### Biochemical assays

Insulin, glucagon, glycogen, and TAG were quantified using Ultra Sensitive Mouse Insulin ELISA Kit (Crystal Chem, 90080), Mouse Glucagon ELISA Kit (Crystal Chem, 81518), Glycogen Assay Kit (BioVision, K646-100), and Triglyceride Quantification Colorimetric/Fluorometric Kit (Sigma, MAK266), following manufacturers’ instructions.

### Corticosterone quantification

A mixture of 1.5 ml methanol and 40 μl corticosterone-d8 internal standard (Santa Cruz Biotechnology, sc-396031) was added into 2 ml Eppendorf tubes, each containing a metal bead. Liver (∼50 mg) samples were added and homogenized using a Qiagen TissueLyser II at 30/s for 30 s. Homogenates were centrifuged 10 min at 12,000*g* and at 4 °C. Supernatants were transferred to round bottom glass tubes and methanol was evaporated under gentle N_2_ streams. Precipitates were resuspended in 2 ml of 0.2 M acetate buffer (pH 5.0) and vortexed 20 s. Ten milliliters of methyl *t*-butyl ether were added. Tubes were vortexed 20 s and centrifuged at 3 min 2000*g*. The upper organic phases were transferred to a new round bottom glass tube and evaporated under gentle N_2_ streams. Precipitates were resuspended in 40 μl methanol. One microliter was injected into HPLC-APCI/MS/MS.

Corticosterone was resolved *via* reverse-phase chromatography with an Agilent 1290 system equipped with a binary pump, column compartment, and autosampler. The column compartment was maintained at 40 °C. Samples were kept in the autosampler at 10 °C. Separation was achieved with an analytical Ascentis Express RP-Amide column (100 × 2.1 mm, 2.7 μm, Sigma Aldrich, 53913-U) at a flow rate of 0.4 ml/min. Mobile phases were (A) 0.1% formic acid in water; (B) 0.1% formic acid in methanol. The following gradient was applied over 25 min: 0 to 2 min, 40% B; 2 to 15 min, 40 to 95% B; 15 to 20 min, holding at 95% B; 20 to 23.5 min, 95 to 40% B; 23.5 to 25 min, back to 40% B and reequilibrating for 1.5 min.

Analytes were detected with a Sciex API-4000 triple-quadrupole mass spectrometer in positive atmospheric pressure chemical ionization mode. Analyst version 1.6 software controlled the instrument, which was operated in the multiple reaction monitoring mode. Mass transitions to produce optimum sensitivity were determined by injecting 1 pmol standards (corticosterone: m/z 347.4 → 329.5, corticosterone-d8: m/z 355.7 → 337.7). Optimized MS variables were as follows: curtain gas, 10 psig; collision gas, 7 psig; ion source gas 1, 70 psig; nebulizer current, 3 μA; source temperature, 350 °C; declustering potential, 55 V; entrance potential, 10 V; collision exit potential, 5 V. Optimized collision energy value was 25 eV.

### Data presentation and statistics

Data are means ± SD. Two-tailed unpaired nonparametric Mann-Whitney tests compared data between two groups. False discovery rate (1%)-adjusted *p*-values were reported for correcting multiple comparisons. Significance values are: ∗*p* < 0.05, ∗∗*p* < 0.01, and ∗∗∗*p* < 0.001. Western blot band density was measured by integrating areas under curves using ImageJ 1.53. Statistical testing was done using GraphPad Prism 9.5.0.

## Data availability

All data are contained within the manuscript.

## Supporting information

This article contains supporting information.

## Conflict of interest

The authors declare that they have no known competing financial interests or personal relationships that could have appeared to influence the work reported in this paper.
