# Supplementary material for: Cyp26a1 supports postnatal retinoic acid homeostasis and glucoregulatory control
Source: J Biol Chem. 2023 Apr 1;299(5):104669. doi: 10.1016/j.jbc.2023.104669 (PMC10176252; doi:10.1016/j.jbc.2023.104669)
Supplement: Supporting information [file mmc1.docx]

**Supporting Information**

***Cyp26a1* supports post-natal retinoic acid homeostasis and glucoregulatory control**

Hong Sik Yoo, Michael A. Cockrum, and Joseph L. Napoli

Graduate Program in Metabolic Biology, Nutritional Sciences and Toxicology, UC-Berkeley

**Supporting Figure 1**. Quantification of liver retinyl esters (A) and retinol (B) by LC/UV, and serum RA by LC/MS/MS. Red circles, 16 hr fasted. Black squares, 16 hr fasted + 6 hr re-fed.


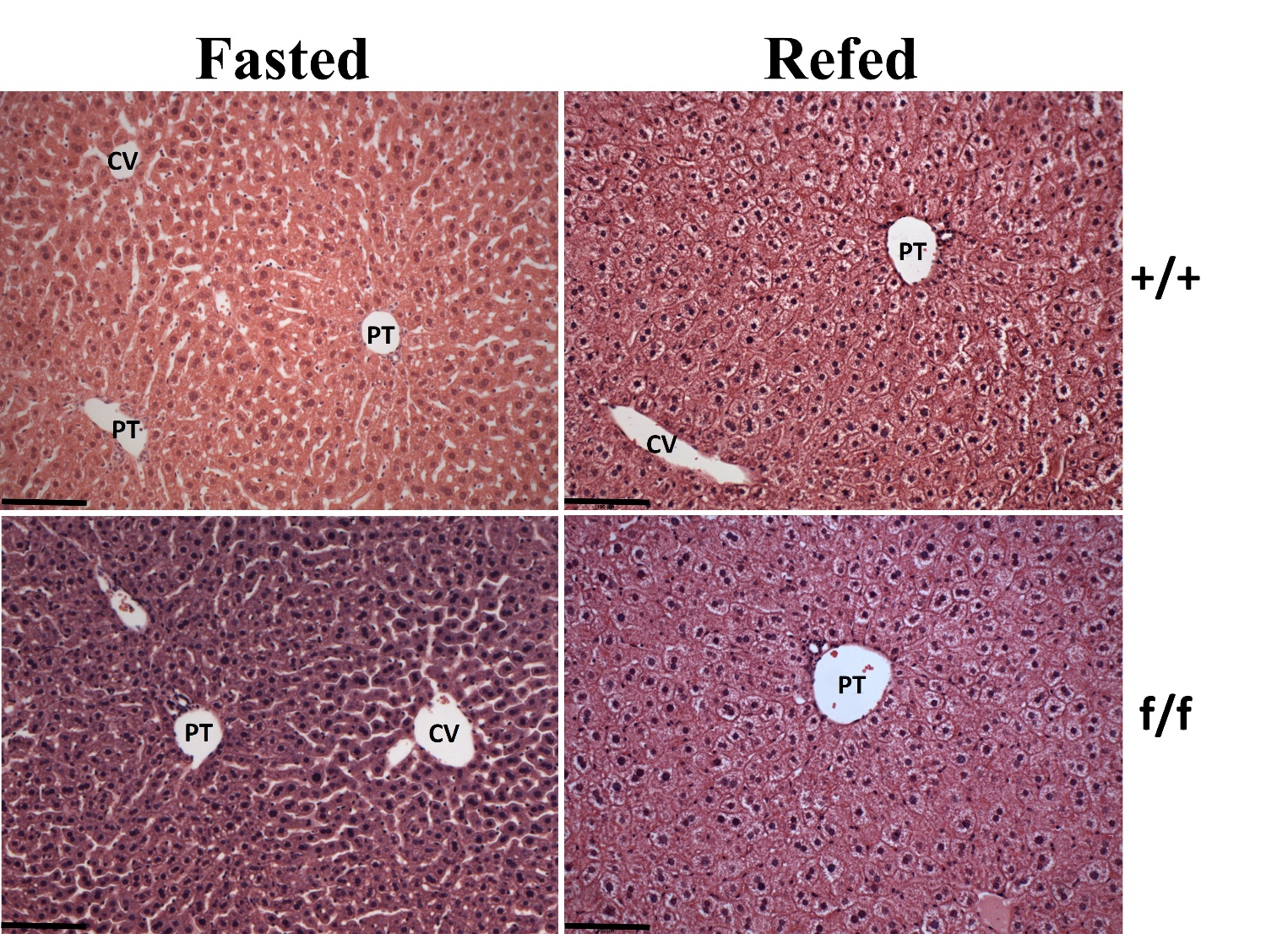


**Supporting Figure 2**. H&E-stained liver sections. CV: central vein, PT: portal triad. +/+: *Cyp26a1* wild-type Cre+. f/f: *Cyp26a1* homozygous floxed Cre+. Scale bars: 100 µm.

**Supporting Figure 3.** Liver glycogen.


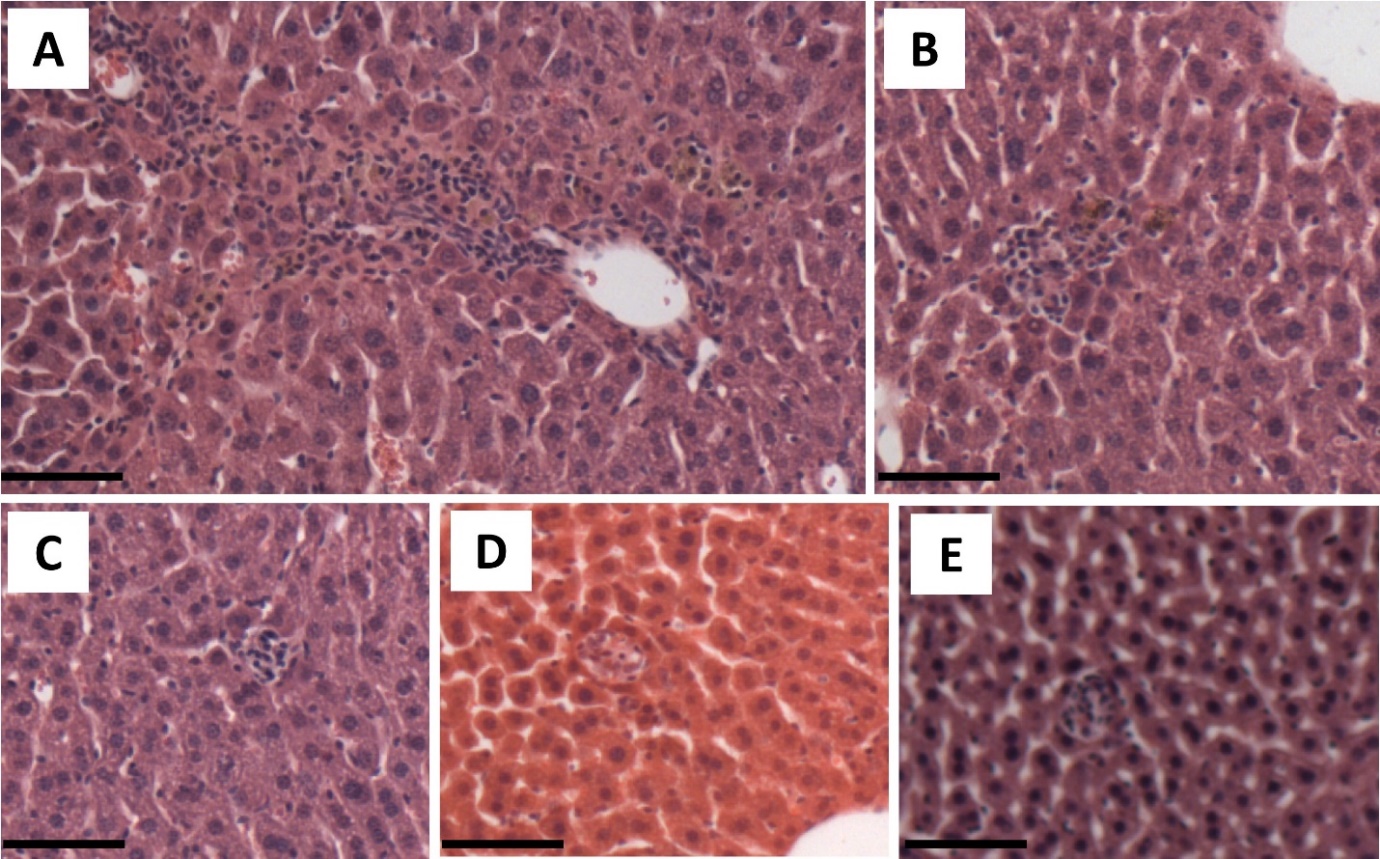


**Supporting Figure 4.** Inflammation foci on H&E-stained liver sections. *A-B*, *Cyp26a1* homozygous floxed Cre+. *C-E*, *Cyp26a1* wild-type Cre+. Scale bars: 50 µm.

**Supporting Figure S5.** Glucagon and insulin values. N = 5-15 mice per genotype per dietary condition.

**Supporting Figure S6.** *Gys2* mRNA (n = 5-15 mice).

**Supporting Table 1.** Numerical values for p-Pygl signals in Fig. 3F.

| **p-Pygl adjusted by β-actin** | | | | **p-Pygl relative to WT fasted +/+ (= 1)** | | | |
| --- | --- | --- | --- | --- | --- | --- | --- |
| **+/+** | | **f/f** | | **+/+** | | **f/f** | |
| **Fasted** | **Refed** | **Fasted** | **Refed** | **Fasted** | **Refed** | **Fasted** | **Refed** |
| 1.1259 | 0.6888 | 0.8303 | 0.7628 | 0.8827 | 0.5400 | 0.6510 | 0.5980 |
| 1.1772 | 0.7660 | 1.3898 | 1.1654 | 0.9230 | 0.6006 | 1.0896 | 0.9136 |
| 1.4325 | 0.8737 | 1.1182 | 0.9110 | 1.1231 | 0.6850 | 0.8766 | 0.7142 |
| 1.3665 | 0.7669 | 1.1635 | 1.2611 | 1.0713 | 0.6012 | 0.9123 | 0.9887 |

Average 1.0 0.6 0.88 0.8

**Supporting Table 2.** qPCR primers.

| Gene | Vendor | ID |
| --- | --- | --- |
| *Cyp26a1* | Integrated DNA Technologies | Mm.PT.58.10791878 |
| *G6pc* | Thermo Fisher Scientific | Mm00839363_m1 |
| *Gck* | Thermo Fisher Scientific | Mm00439129_m1 |
| *Gusb* | Integrated DNA Technologies | Mm.PT.39a.22214848 |
| *Gys2* | Integrated DNA Technologies | Mm.PT.58.42540064 |
| *Pck1* | Integrated DNA Technologies | Mm.PT.58.11992693 |
| *Pdk4* | Integrated DNA Technologies | Mm.PT.58.9453460 |
| *Pygl* | Integrated DNA Technologies | Mm.PT.58.5581132 |
